# Galangin’s Therapeutic Potential in Retinoblastoma: Inducing Apoptosis and Modulating RB Protein and Some MicroRNAs

**DOI:** 10.34172/apb.025.46369

**Published:** 2025-12-20

**Authors:** Maryam Zamani Sani, Mohammad Mirzaei, Ali Mota, Effat Alizadeh, Maryam Ghasemi, Elmira Aboutalebi Vand Beilankouhi, Niloufar Kheradi, Mohammad Rahmati

**Affiliations:** ^1^Immunology Research Center, Tabriz University of Medical Sciences, Tabriz, Iran; ^2^Department of Clinical Biochemistry and Laboratory Medicine, Tabriz University of Medical Sciences, Tabriz, Iran; ^3^Department of Ophthalmology, Nikoukari Eye Hospital, Medical School, Tabriz University of Medical Sciences, Tabriz, Iran; ^4^Department of Medical Biotechnology, Faculty of Advanced Medical Sciences, Tabriz University of Medical Sciences, Tabriz, Iran; ^5^Department of Animal Biology, Faculty of Natural Sciences, University of Tabriz, Tabriz, Iran; ^6^Molecular Medicine Research Center, Tabriz University of Medical Sciences, Tabriz, Iran

**Keywords:** Galangin therapy, Apoptosis, MicroRNAs, Retinoblastoma

## Abstract

**Introduction:**

Retinoblastoma is a type of cancer that develops in the retina, mainly affecting infants and young children under five years old. Research indicates that microRNAs (miRNAs) can either promote or suppress cancer, significantly impacting its development. Galangin exhibits potential anticancer effects, but further research is necessary to confirm its benefits.

**Methods:**

In this study, the MTT assay was used to assess cell viability. Changes in microRNA expression were analyzed using real-time PCR. The apoptotic effect of galangin was determined using flow cytometry. Changes in RB protein expression due to galangin treatment were determined using Western blotting. A t-test and 2-ΔΔCT were used for data analysis. Statistical significance was considered at *P*<0.05.

**Results:**

An IC50 of 13.08 μM was determined for the 48-hour MTT assay. Galangin treatment decreased the expression of miR-106a-5p by about 1.41-fold (*P* value: 0.0375). An about 1.11-fold increase was also observed for mir-519a-3p (*P* value: 0.0207). The change in miR-192-5p expression was insignificant (*P* value: 0.4677). A decrease of approximately 1.87-fold was also shown for miR-21-5p (*P* value: 0.0072). The apoptotic effect of galangin at the IC50 concentration and a dose of 26 µM was 34.30% and 26.70%, respectively. RB protein expression increased 2.66-fold approximately (*P* value: 0.0045).

**Conclusion:**

Our study showed that galangin has an apoptosis-inducing effect in Y79 cell lines. The apoptosis-inducing effect can be dose-dependent and can be reduced by increasing the dose. The increase in RB protein due to galangin treatment is accompanied by an increase in apoptosis. Galangin also affects the expression of miR-106a-5p, miR-519a-3p, and miR-21-5p.

## Introduction

 Retinoblastoma is a cancer that arises in the retina and typically affects infants or young children, particularly those under 5 years old. This cancer represents a frequent type of eye tumor in children, making up about 2.5% to 4% of all cancers found in childhood.^1^ Studies have shown that miRNAs can act as either oncogenes or tumor suppressors, influencing the progression or inhibition of cancer. They play a crucial role in the carcinogenesis process.^2^ So, modulating the expression of miRNAs can restore the balance and inhibit cancer progression. Today, researchers are focusing on the clinical application of miRNAs, which can be used as a therapeutic tool to enhance the response to standard therapies.^3^

 Osteonecrosis, pulmonary toxicity, neurologic/psychological effects, gastrointestinal organ effects, kidney/urinary bladder effects, heart disease, immunologic system effects, hormonal/reproductive effects, and second cancers are late side effects of cancer treatment.^4^ Therefore, alternative therapies that specifically target cancer cells should be employed to minimize side effects.^5^ Scientists’ curiosity about the effects of nature on human health has led to extensive research on flavonoids, a family of polyphenolic phytochemicals that are naturally found in plants. Evidence has shown that flavonoids have antioxidant, antidiabetic, anti-aging, anti-allergic, antiplatelet, anti-inflammatory, and anticancer activities.^6^

 Galangin, a substance found in Alpinia officinarum, commonly known as lesser galangal, is a natural flavonoid.^7^ Galangin has antioxidant, anti-obesity, antiviral, antimicrobial, and anti-inflammatory activities. Galangin also exhibits anticancer properties, which require extensive research in this field to confirm.^8^ The objective of this research is to examine the impact of galangin on retinoblastoma cells (Y79 cell line), especially its effect on apoptosis induction and changes in RB protein expression. Additionally, the change in the expression of specific miRNAs upon exposure to galangin has been investigated as a potential molecular biomarker for the diagnosis and follow-up of retinoblastoma. The results of this study can be considered an essential step towards introducing galangin as an alternative treatment option and utilizing miRNAs as biomarkers in the diagnosis and evaluation of retinoblastoma.

## Materials and Methods

###  Conditions for Cell Culture 

 Y79 cells were cultured in RPMI 1640 + 10% FBS and incubated with 5% CO_2_ at 37°C.

###  Cell Survival Percentage

 Y79 cells were treated with concentrations of 0, 10, 20, 40, 80, and 160 μM galangin and incubated for 24, 48, and 72 hours. After that, they were incubated again for 4 hours with the addition of 3-(4,5-dimethylthiazol-2-yl)-2,5-diphenyltetrazolium bromide (MTT). The dye was dissolved in DMSO. The plate was read using a microtiter plate reader at a wavelength of 570 nm. A blank was used as a control.

###  Real-Time PCR

 RNA extraction was performed using 1 ml of Trizol (Yekta Tajhiz Azma, Iran). Then, cDNA synthesis (EXIQON kit, Denmark) was performed from the extracted RNAs according to the manufacturer’s instructions. This kit employs the use of LNA-based chemistry as its mechanism. The protocol to perform the method is presented in Table 1. Following this, real-time RT–PCR amplification was carried out using a SYBR Green qPCR master mix (Yekta Tajhiz Azma, Iran). The 2^-ΔΔCT^ method was employed to compare the expression of miRNAs in a treatment group with an untreated control, using a reference gene (U6) for normalization. Table 2 lists the primer sequences of the miRNAs.

**Table 1:** The protocol of cDNA synthesis.

**Step **	**Function**	**Procedure**
1	Preparation of RNA Samples and Reaction Components	The nuclease-free water was used to dilute the RNA samples to a final concentration of 5 ng/µL. The 5 × Reaction Buffer and nuclease-free water were thawed slowly and put on ice. After the mixing by vortexing, the RNA spike-ins were reconstituted according to the appropriate RNA spike-ins protocol and incubated on ice for 15–20 minutes. Just before use, the Enzyme Mix was taken out of the freezer, flicked gently to mix, and then placed on ice. All the reagents were spun down before use.
2	Reverse Transcription Procedure	The reverse transcription (RT) reaction was established for first-strand cDNA synthesis by employing 5 × Reaction Buffer, nuclease-free water, Enzyme Mix, and by the manufacturer's guide, the use of RNA spike-ins. In a 10 µL reaction, 2 µL of 5 × Reaction Buffer, 4.5 µL of nuclease-free water, 1 µL of Enzyme Mix, 0.5 µL of synthesized RNA spike-in, and 2 µL of template RNA (5 ng/µL) were combined. The reagents were gently mixed by vortexing or by pipetting in an attempt to homogenize and spun down. The cDNA synthesis reaction mixtures were incubated for 60 minutes at 42°C, then the reverse transcriptase was inactivated by heating at 95°C for 5 minutes. The cDNA was then cooled to 4°C and either stored at 4°C or frozen for future use.

**Table 2:** Primers and their sequences.

**Name**	**Sequence (5’→3’)**
Fp hsa-miR-106a-5p	AACACGCAAAAGTGCTTACAGT
Fp hsa-miR-519a-3p	AACACGCAAAGTGCATCCTTT
Fp hsa-miR-192-5p	AAGCGACCCTGACCTATGAAT
Fp hsa-miR-21-5p	AAGCGACCTAGCTTATCAGACT
Fp hsa-U6	AACAAGTTGGAACGATACAGAGAAG
RP-Universal	GTCGTATCCAGTGCAGGGT

###  Apoptosis Assay

 Each dose of galangin in the treatment group was tested independently in two biological samples to confirm the consistency of its results. The assessment of Y79 cell apoptosis was conducted utilizing the Annexin V-FITC Apoptosis Detection Kit (Immunostep, Spain), in accordance with the manufacturer’s guidelines. Following a 48-hour treatment period, Y79 cells were collected. Subsequently, the cell pellet was isolated, and the cells were washed with PBS before being resuspended in a binding buffer containing FITC-conjugated Annexin V and PI. After a 15-minute incubation in the dark, the percentage of cell apoptosis induced by galangin treatment was quantified.

###  Western Blotting

 The experiment was conducted independently on two biological samples and in duplicate to confirm the results. To measure the apoptosis-related protein, RB, Western blot analysis was performed on 10^6^ cells. Following cell lysis and centrifugation, the proteins were separated using gel electrophoresis and then transferred to a membrane. The membranes were then blocked and incubated with primary antibodies. Washing was performed, and incubation was repeated with secondary antibodies. Immunoreactive bands were detected using ECL solution and quantified using ImageJ software. Primary and secondary antibodies are shown in Table 3.

**Table 3:** Type of antibodies in western blotting.

**Primary Antibodies**	**Secondary Antibodies**
(Cat. no: sc-102)	m-IgGκ BP-HRP (Cat. no: sc-516102; Santa Cruz Biotechnology, Inc.)

###  Statistical Analysis

 The study groups, the control group, and the treatment group were selected. T-test and 2^-ΔΔCT^ were used to analyze the data using GraphPad software version 8. Statistical significance was considered at *P* < 0.05.

## Results

###  MTT Assay

**Figure 1:**
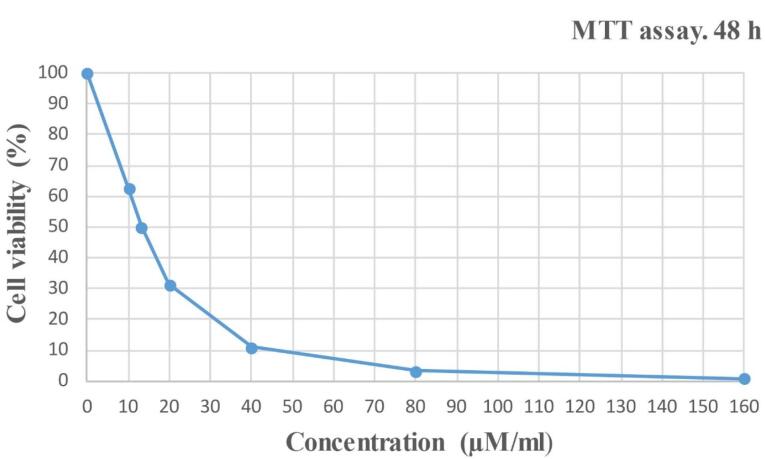
Concentration- Cell viability (%) plot

 After reading the OD of the wells at 570 nm via a microplate reader (Immunoskan MS, Labsvstems, Finland), the half-maximal inhibitory concentration (IC50) calculation was performed using GraphPad Prism software. IC50 value was calculated to be 13.08 µM. The equation of the curve was determined to be Y = 0 + 0.996/ 1 + (x / 13.08)^1.891^. The 95% confidence interval (95% CI) values are shown in Table 4. The concentration-cell viability (%) plot is shown in Figure 1.

**Table 4:** 95% CI values.

**95% CI (profile likelihood)**	
LogIC50	1.081 to 1.149
HillSlope	-2.182 to -1.636
IC50	12.06 to 14.10

###  Galangin Affects the Expression of mir-106a-5p, mir-519a-3p, mir-192-5p, and mir-21-5p.

**Figure 2:**
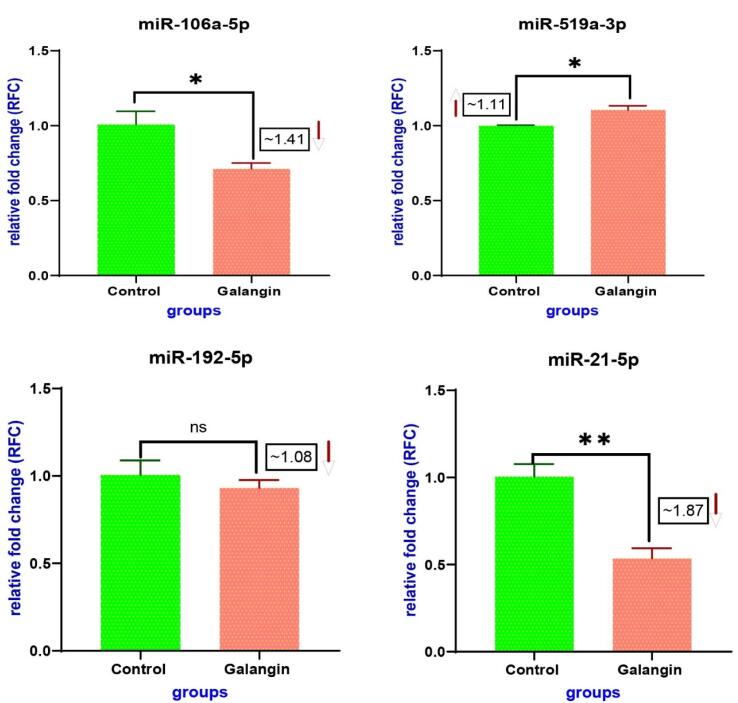
Fold change of miRNAs. *P* value of mir-106a-5p: 0.0375, *P* value of mir-519a-3p: 0.0207, *P* value of mir-192-5p: 0.4677, and *P* value of mir-21-5p: 0.0072

 The real-time PCR results are shown below (Figure 2). The relative fold change (RFC) for each microRNA is presented alongside the mean, standard deviation, and standard error of the mean. A significance threshold of *P* < 0.05 was established. Mean, Std. Deviation, and Std. The error of the Mean of miR-106a-5p was calculated as 1.008, 0.1533, and 0.08853 in the control group and 0.7125, 0.06673, and 0.03852 in the treatment group, respectively. Mean, Std. Deviation, and Std. The error of the Mean of miR-519a-3p was calculated as 1.000, 0.006932, and 0.004002 in the control and 1.105, 0.04867, and 0.02810in the treatment, respectively. Mean, Std. Deviation, and Std. The error of the Mean of miR-192-5p was calculated as 1.006, 0.1420, and 0.08197 in the control group and 0.9310, 0.07982, and 0.04608 in the treatment group, respectively. Mean, Std. Deviation, and Std. The error of the Mean of miR-21-5p was calculated as 1.005, 0.1248, and 0.07205 in the control group and 0.5352, 0.1020, and 0.05888 in the treatment group, respectively.

###  Galangin Induces Apoptosis in Y79 Retinoblastoma Cells

 The flow cytometry assay was conducted to examine the apoptotic effects of galangin on the Y79 retinoblastoma cell line. The following steps were performed for flow cytometric analysis:

1) The gate strategy was determined.
2) Single cells were separated, and doublet and aggregate cells were removed to improve data quality, and
3) Labeling was done for each axis.

**Figure 3:**
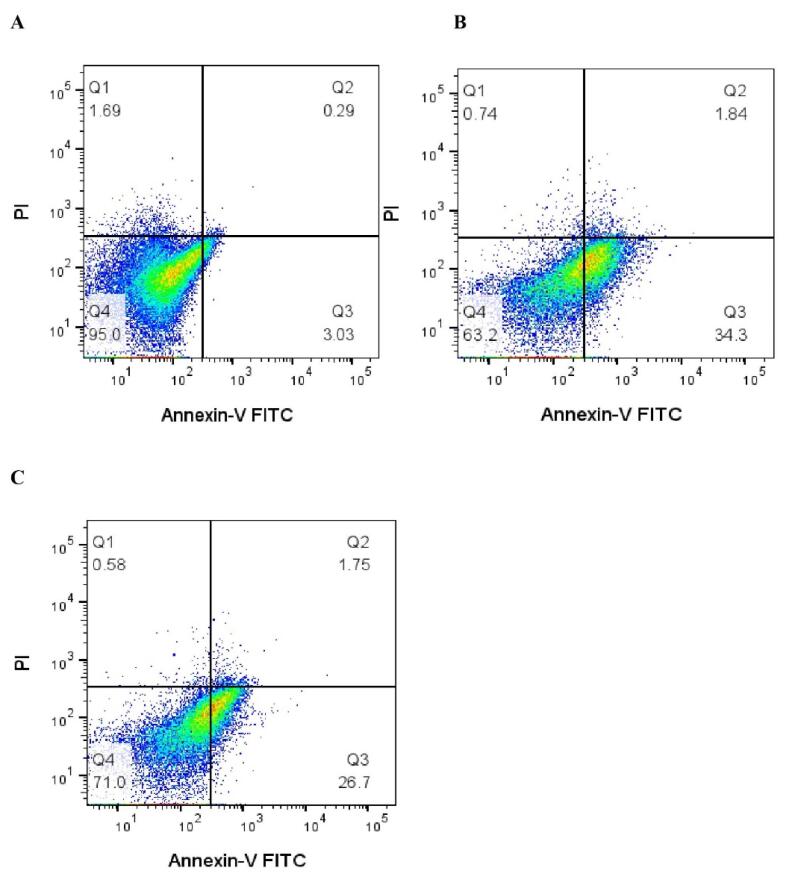
(A) Control. (B) Cell apoptosis with treatment of 13.08 µM galangin. (C) Cell apoptosis with treatment of 26 µM galangin

**Figure 4:**
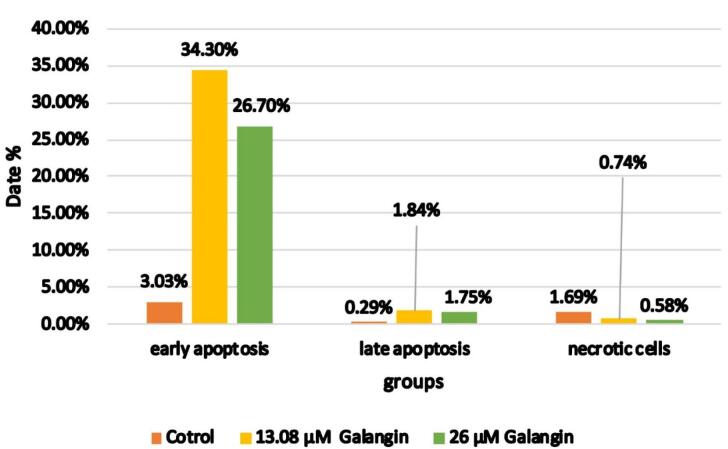
Graph of apoptosis percentage for control, treatment of 13.08 µM galangin, and treatment of 26 µM galangin

The final flow cytometric analysis plot for apoptosis for the control and treated samples with concentrations of 13.08µM and 26 µM is shown below (Figure 3). A graph is also provided to compare apoptosis between samples in Figure 4.

###  Galangin Increases the Expression of the RB Protein

**Figure 5:**
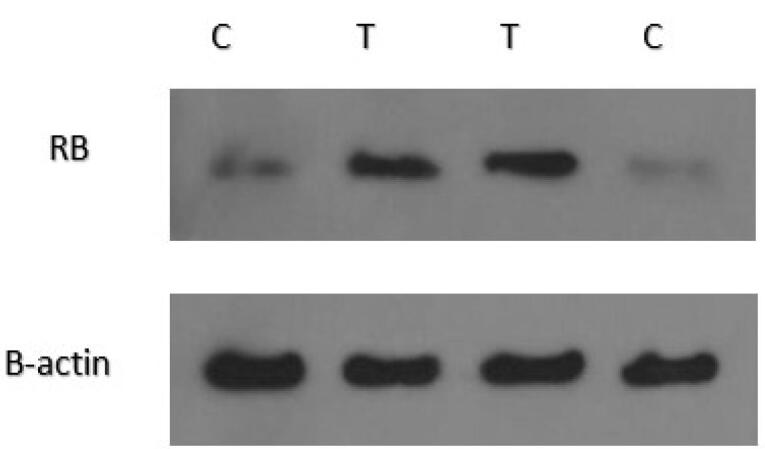
Change in RB protein expression in control and treated samples at IC50 concentration

**Figure 6:**
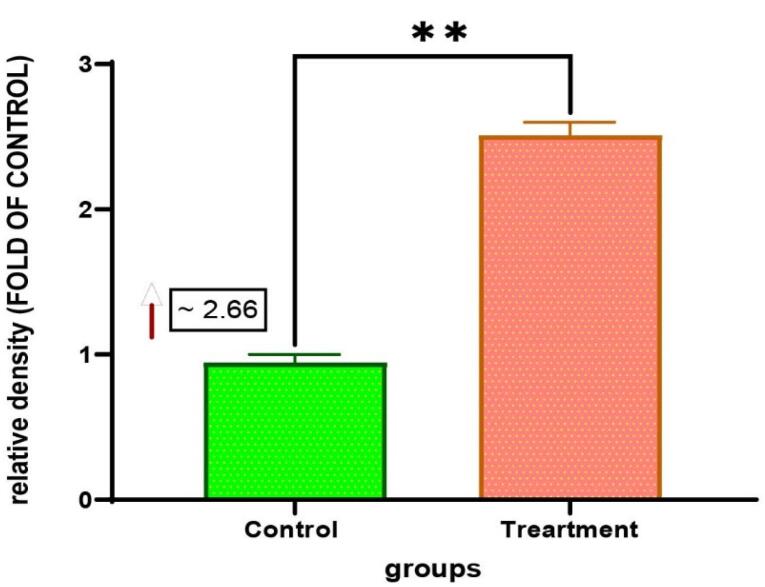
Change in RB protein expression in control and treated samples at IC50 concentration with *P*-value: 0.0045

 A Western blot test was performed to determine the expression of the RB protein. RB protein expression in Y79 cells treated for 48 hours with galangin increased compared to the control by almost 2.66 times and significantly with *P* < 0.05. To clarify the band size specificity, we employed the SuperSignal® Molecular Weight Protein Ladder (Cell Signaling #84785), which comprises eight distinct bands (20–150 kDa). The Rb protein with a molecular weight of approximately 110 kDa was detected. Figures 5 and 6 show the results of the Western blot test below.

## Discussion

 About 300 children in the United States are diagnosed with retinoblastoma, the most common intraocular cancer in children, each year.^9^ Radiation therapy, chemotherapy, and surgery are traditional cancer treatments with non-specific approaches.^10^ Modern approaches to cancer treatment include immunologically targeted therapies and molecularly targeted therapies. Immune checkpoint inhibitors and chimeric antigen receptor (CAR) T-cell therapy are among these therapies.^11^ Anti-angiogenic approaches, stem cell therapies, and hormone-based therapy are other emerging treatments.^12^ While current treatments have improved eye salvage and survival rates, they also have many side effects, including an increased risk of secondary cancers.^13^ Children under one year of age have high long-term risks from exposure to ionizing radiation.^9^ Targeted therapies with reduced toxicity are becoming possible due to recent advances in the analysis and study of RB1 mutations.^14^ Due to available resources, germline mutation status, and disease staging, treatment decisions are tailored to individual needs.^15^ Considering the complications that may result from the treatment, an alternative treatment with greater safety should be sought.

 Galangin, a non-toxic phytochemical, has therapeutic applications and exerts its anticancer effects through antiangiogenic and apoptosis induction properties. Galangin, when combined with other compounds or gold nanoparticles, exhibits therapeutic effects against various types of cancer and can be considered an alternative cancer therapy.^16^ Galangin can exhibit potent anticancer properties through multiple mechanisms. Galangin can decrease the expression of Bcl-2 and increase the expression of Bax and Cyt-c. Galangin can also inhibit the invasion and migration of cancer cells, as well as suppress the expression of certain proteins involved in the PI3K/AKT/mTOR signaling pathway.^17^ Galangin degrades β-catenin and inhibits tumor cell proliferation. This process leads to a decrease in c-myc and cyclin D1 expression.^18^ Galangin also exerts its chemopreventive effect through its ability to increase oxidative and carbonyl stress and inhibit glyoxalase-1 through redox signaling.^19^ There are many different mechanisms through which galangin exerts its anti-cancer effects, only a few of which were mentioned.These diverse mechanisms make galangin a promising candidate for cancer treatment.

 The IC50 value indicates the amount required to inhibit a process by half and is a crucial measure of a drug’s efficacy, widely used in pharmacological research.^20^ A longer incubation period is required to assess cytotoxicity.^21^ To achieve reliable results in assessing drug resistance and chemosensitivity in cancers, it is necessary to consider an optimal incubation time of 48-96 hours in studies.^22^ Previous studies have demonstrated that using the 48-hour MTT assay in acute myeloid leukemia enhances the assay’s sensitivity, and the in vitro data are closely correlated with the in vivo data.^23^ In this study, MTT assays were conducted at 24, 48, and 72 hours. In the 24-hour MTT and 72-hour MTT assays, we did not obtain acceptable and significant results. The results were significant in the 48 h MTT. Additionally, considering the above, the remaining work was conducted using the IC50 obtained from the 48 h MTT assay (IC50: 13.08 µM).

 In cancer patients, miRNAs are dysregulated, making them a particular focus of interest in the field of research. They play a vital role in metastasis, tumor growth, and even cancer initiation and are promising in cancer therapy. miRNAs may be utilized in the future for cancer diagnosis and treatment.^24^ In cancer patients, miRNAs can be used as reliable biomarkers. Their expression levels can also be used to stage tumors. MiRNAs are detected in body fluids and can be used for non-invasive investigations.^25^ Dysregulation of miRNAs is associated with the prognosis, prediction, diagnosis, progression, tumorigenesis, and treatment of most cancers. Therefore, they have become a therapeutic target in cancer research.^26^

 One of the most significant childhood cancers is retinoblastoma. This disease is caused by inactivation of the RB (tumor suppressor) gene, and early detection is crucial for its treatment.^27^ MiRNAs have a significant impact on treatment resistance and the development of resistance to RB because they regulate various cellular functions.^28^ The prognosis and diagnosis of RB are closely linked to its expression profile.^29^ By identifying several circulating and tissue-specific miRNAs in RB, researchers have demonstrated that miRNAs have the potential to be used as prognostic, diagnostic, and even therapeutic markers.^27^

 In this study, the expression of miR-106a-5p, miR-519a-3p, miR-192-5p, and miR-21-5p was investigated in retinoblastoma cells treated with galangin for 48 hours and in cells without treatment.

 miR-106a-5p promotes cell migration and invasion in colorectal, gastric, and breast cancer by targeting TGFBR2 (transforming growth factor-β receptor 2), FAS, and ZBTB4 (zinc finger and BTB domain-containing 4). However, in glioma, targeting E2F1 (E2F transcription factor 1) induces apoptosis and inhibits cell proliferation.^30^ In kidney tumor cells, increased levels of miR-106a-5p reduce the expression of vascular endothelial growth factor A (VEGFA) and, by inhibiting cell proliferation, lead to cell death. MiR-106a-5p is downregulated in osteosarcoma, ovarian cancer, and astrocytoma.^31^ Therefore, according to the studies, the function of miR-106a-5p depends on the type of tumor and has two roles: oncogenic and tumor suppressor. In this examination, mir-106a-5P was reduced by about 1.41-fold and showed an oncogenic role (*P* Value: 0.0375).

 MiR-519a-3p promotes resistance to cell death in breast cancer, enabling cancer cells to evade destruction by the immune system. It can also prevent tumor cells from being recognized by natural killer (NK) cells, thereby influencing the tumor microenvironment. miR-519a-3p also makes tumor cells resistant to apoptosis.^32^ Gastric cancer (GC) exosomal miR-519a-3p induces M2-like macrophage-mediated angiogenesis along with liver metastasis. Targeting it can prevent the formation of premetastatic gaps and provide insights into antitumor metastasis therapy. Therefore, miR-519a-3p may provide insights into antitumor metastasis therapy.^33^ MiR-519a-3p expression is downregulated in glioblastoma. circNUP98 represses miR-519a-3p, resulting in increased cell proliferation.^34^ In this investigation, mir-519a-3p increased approximately 1.11-fold and has a suppressive role (*P* Value: 0.0207).

 The expression levels of miR-192-5p are elevated in healthy individuals compared to those with lung cancer and bone metastasis, and this miRNA plays a role in inhibiting cancer progression by targeting TRIM44.^35^ MiR-192-5p is observed to be downregulated in malignant tumors, and its overexpression has been shown to suppress tumorigenesis. In the context of osteosarcoma, miR-192-5p plays a significant role in inhibiting tumorigenesis by targeting ubiquitin-specific protease 1 (USP1). Consequently, miR-192-5p may serve as a promising biomarker for this condition. Furthermore, the miR-192-5p/USP1 pathway could be considered a novel therapeutic target.^36^ MiR-192-5p is involved in various cancers, including liver, breast, bladder, and colon cancers. In breast cancer, it increases the sensitivity of cells to doxorubicin and inhibits the growth of bladder cancer. In papillary thyroid carcinoma cells, it inhibits the migration, invasion, and even proliferation of tumor cells through SH3RF3.^37^ In this study, the change in miR-192-5p’s expression was not significant, with a *P*-value of 0.4677.

 Decreased miR-21 expression is observed in samples from healthy individuals compared to those from patients with squamous cell carcinoma of the conjunctiva (SCCC).^38^ Increased expression of miR-21-5p has been observed in uveal melanoma (UM) and has been shown to increase metastatic risk.^39^ In hepatocellular carcinoma (HCC), the expression of miR-21-5p is increased. miR-21-5p controls the expression of PTEN (phosphatase and tensin homolog) and activates hypoxia-inducible factor 1 (HIF-1) and HIF-2. Thus, it promotes tumor growth and spread by preventing tumor cell apoptosis.^40^ The induction of epithelial-to-mesenchymal transition (EMT) in endometrial cancer is facilitated by the targeting of SRY-box 17 by miR-21-5p.^41^ In colorectal cancer cells, the increased levels of miR-21-5p promote the self-organization of multicellular tumor spheroids that rely on E-cadherin.^42^ In HCC, the inhibitory effect of cisplatin (DDP) is reduced by the overexpression of miR-21-5p. FAS ligand (FASLG) is targeted by miR-21-5p and desensitizes HCC cells to DDP treatment.^43^ In this research, miR-21-5p was found to be decreased by approximately 1.87-fold and was associated with an oncogenic role (*P* value: 0.0072).

 In this study, we investigated the apoptotic effect of galangin at concentrations of 13.08 μM and 26 μM on retinoblastoma cells that were treated with galangin for 48 hours.

 Several studies have demonstrated that galangin exhibits an apoptosis-inducing effect in cancer cells. For example, galangin induces apoptosis in colon cancer cells (HT-29 and HCT-15), leading to mitochondrial dysfunction, caspase activation, and the release of apoptosis-inducing factor (AIF).^44^ In renal cancer cells, galangin enhances TRAIL-induced apoptosis by reducing the expression of anti-apoptotic proteins.^45^

 Conversely, galangin exhibits protective effects against apoptosis under certain conditions. For example, it suppresses apoptosis in bone marrow mesenchymal stem cells, promoting cell survival.^46^ Galangin reduces cerebral infarction and neurodegeneration in ischemic stroke models. Galangin exerts this effect by inhibiting the apoptotic pathway, reducing mitochondrial dysfunction, and improving regional cortical blood flow.^47^ Galangin also protected keratinocytes against UVB-induced apoptosis and oxidative stress by restoring mitochondrial polarization and inhibiting the formation of free radicals.^48^ Galangin activates the PI3K/AKT pathway, reduces oxidative stress, and inhibits apoptosis in liver ischemia-reperfusion injury.^49^ Therefore, the balance between pro-apoptotic and anti-apoptotic signals is crucial, and the ability of galangin to alter this balance could lead to the induction or inhibition of apoptosis.

 In our study, as shown, the rates of primary apoptosis in cells treated for 48 hours with doses of 13.08 µM and 26 µM were 34.30% and 26.70%, respectively. The paradoxical effect of reducing cell apoptosis with increasing galangin dose can be generally attributed to several factors. Here we mention some of these cases:

 Galangin has biphasic effects at higher concentrations. At lower doses, it induces apoptosis,^50^ and at higher doses, it triggers cellular stress responses and activates survival pathways to counteract apoptosis. This phenomenon is common in pharmacology, where drugs have different effects based on their concentration. Higher doses of the drug induce survival pathways and cellular stress responses through apoptosis and oxidative stress. In renal models, galangin reduces renal damage and enhances cell survival under stress conditions by activating the SIRT1/Nrf2/HO-1 pathway, thereby improving cellular defense against apoptosis and oxidative stress.^51^ For cellular antioxidant responses, the SIRT1/PGC-1α/Nrf2 signaling pathway plays an important role. Galangin promotes this pathway. This mechanism helps to enhance cell survival, particularly in skin cells exposed to H2O2 and UV, and reduces oxidative damage.^52^ Increased cell proliferation can result from increased apoptosis. Cells that have escaped apoptosis are stimulated to proliferate rapidly, thereby maintaining the cell population.^53^ Apoptosis has a dual role. That is, it is both a barrier to cancer and can promote cancer progression. Apoptotic cells produce potent growth-promoting signals that can facilitate tumor growth. One of these signals is prostaglandin E2, which is dependent on caspase-3. This phenomenon is called “Phoenix Rising”.^54^ Many treatments used to get rid of cancer activate the apoptosis pathway in tumor cells. These treatments sometimes select for more resilient cancer cells. The result of this treatment is that the cells survive after treatment and then proliferate rapidly.^54^ Apoptosis enhances resistance to therapy. Apoptosis activates innate immune cells and creates a tumor TME that is pro-oncogenic and prevents cancer cells from being treated. Thus, apoptosis has a “double-edged” role, serving both as a cancer suppressor and a cancer promoter. Another possible explanation for this phenomenon is that apoptosis enhances the vital proliferation required to restore tissue homeostasis, allowing selected clones to expand and grow.^55^

 Unphosphorylated Rb protein represses E2F, leading to the inhibition of cell proliferation. In the G1/S phase of the cell cycle, Rb phosphorylation occurs, releasing E2F. This process promotes cell cycle progression. Rb protein is dephosphorylated by protein phosphatase 1 (PP1) in late M phase, thus restoring its regulatory function. While dephosphorylated Rb is critical for apoptosis, phosphorylated Rb plays roles in cell proliferation and protection.^56^

 In this investigation, we noted a statistically significant increase in RB protein expression (*P* < 0.05) in the cell line treated with the IC50 concentration of galangin compared to the control in retinoblastoma cells. Several factors may contribute to the approximately 2.66-fold increase in RB protein expression. Some of the factors likely to be affected by galangin are discussed below:

 In colon, ovarian, and breast cancer cells, siRNA-mediated knockdown of PNUTS (nuclear phosphatase targeting subunit) results in increased PP1 activity, dephosphorylation of Rb, and induction of apoptosis.^56^ Therefore, one of the factors that increases the expression of the RB protein may be the effect of galangin on the regulation of PP1, followed by the increase of PP1 activity and dephosphorylation of RB. Another possibility is the effect on the protein phosphatase 1-spinophilin (PP1-SPN) complex. PP1-SPN dephosphorylates RB during the G0/G1 transition.^57^ Some treatments, e.g., rosquitin, by inhibiting CDK function, cause RB to be rapidly dephosphorylated. This process suggests a direct relationship between increased phosphatase activity and decreased kinase activity.^58^ The p53 protein is involved in regulating the phosphorylation state of RB in the context of genotoxic stress. This conclusion follows from the fact that accumulation of p53 leads to further dephosphorylation of RB.59 The increase in RB expression could be due to the p53 pathway. This pathway is critical for the process of apoptosis. p53 can affect both the intrinsic pathway, such as Noxa, Puma, and Bax, and the extrinsic pathway, such as Killer/DR5.^60^ Increased p53 expression can lead to increased RB expression and induce apoptosis.^61^ Key regulators of the cell cycle are the RB-E2F target genes. Their expression is promoted by phosphorylation of RB. p53 activates the CDK inhibitor p21/CDKN1A, preventing this process. The components that make up the p53-p21-RB signaling pathway are these elements. The activation of p53 leads to an increase in p21 expression, which in turn causes a decrease in the expression of cell cycle genes.^62^ In MCF-7/S breast cancer cells, a direct relationship exists between the amount of apoptosis and the levels of dephosphorylated RB protein expression. With increasing concentrations of Adriamycin (ADR), the expression of dephosphorylated RB protein increases, and apoptosis is induced.^63^ As a pro-apoptotic factor in radiation-induced apoptosis, RB enhances apoptosis. This enhancement of apoptosis is not mediated by caspase activation. It is therefore likely that the role of RB varies in response to cellular stressors.^64^ The breast cancer cell line MDA-MB-468, which lacks the RB protein, is highly sensitive to low doses of ultraviolet (UV) radiation. The prostate cancer cell line DU-145, which also lacks functional RB, also exhibits a similar apoptotic response.^65^ Apoptosis induced by CDK inhibitors or UV can be blocked by the phosphorylation of specific RB sites and enhanced interaction with Bax, thereby suppressing apoptosis.^57^ In some cancers, sporadic eye tumors, the RB gene is mutated, and in most human cancers, the RB protein is inactive. It is a cell cycle regulator and tumor growth suppressor, but it can inhibit apoptosis and play an oncogenic role in the cell. It plays a general role in cellular homeostasis during cancer development.^66^ RB’s role in apoptosis is still debated, as it can act to promote or inhibit apoptosis depending on the context.^61^

## Conclusion

 Galangin has an apoptosis-inducing effect in Y79 cell lines. The apoptosis-inducing effect can be dose-dependent and can be reduced by increasing the dose. The increase in RB protein due to galangin treatment is accompanied by an increase in apoptosis. Galangin also affects the expression of miR-106a-5p, miR-519a-3p, and miR-21-5p. Of course, it is worth noting that microRNAs have a dual role, which can be either oncogenic or tumor suppressor, depending on the cell or tissue’s conditions and environment. There is a possibility that the expression of the RB gene or microRNAs can be altered due to changes in the upstream mechanisms. In this study, we observed changes in gene expression in response to galangin; however, our investigation does not focus on the mechanism of action of the drug. This study was conducted as an initial exploratory investigation to evaluate the effects of galangin on human retinoblastoma (RB) cells. Although only a single RB cell line was used and no nonmalignant retinal control was included, the untreated RB cells served as an internal control, allowing a valid comparison with the treated group. We acknowledge that the absence of additional RB cell lines, normal retinal cells, and cytotoxic selectivity assessments limits the generalizability of our findings. These factors should be addressed in future research to further validate the therapeutic and biomarker potential of the galangin in RB. Nevertheless, the present results provide important preliminary insights that can guide more comprehensive studies in this area. Therefore, it requires numerous studies with different types of inhibitors to determine precisely which genes or proteins are affected and what functional consequences this change can have. Bioinformatics plays a crucial role in this field. Indeed, numerous bioinformatic and experimental studies are needed to reach a definitive conclusion. Additionally, minimal studies have been conducted in this field, and a thorough study path is required to achieve accurate results.

## Competing Interests

 The other authors report no conflicts of interest.

## Ethical Approval

 The Ethics Regional Committee and the Research Council of the Immunology Research Center, as well as the Research Council of the Faculty of Medicine, Tabriz University of Medical Sciences, with the ethical code IR.TBZMED.VCR.REC.1401.094, approved the above project.
